# Efficacy of psychosocial interventions to reduce alcohol use in comorbid alcohol use disorder and alcohol-related liver disease: a systematic review of randomized controlled trials

**DOI:** 10.1093/alcalc/agad051

**Published:** 2023-08-01

**Authors:** Sofia Hemrage, Eileen Brobbin, Paolo Deluca, Colin Drummond

**Affiliations:** Department of Addictions, National Addiction Centre, Institute of Psychiatry, Psychology and Neuroscience, King’s College London, 4 Windsor Walk, London SE5 8AF, United Kingdom; Department of Addictions, National Addiction Centre, Institute of Psychiatry, Psychology and Neuroscience, King’s College London, 4 Windsor Walk, London SE5 8AF, United Kingdom; Department of Addictions, National Addiction Centre, Institute of Psychiatry, Psychology and Neuroscience, King’s College London, 4 Windsor Walk, London SE5 8AF, United Kingdom; Department of Addictions, National Addiction Centre, Institute of Psychiatry, Psychology and Neuroscience, King’s College London, 4 Windsor Walk, London SE5 8AF, United Kingdom

**Keywords:** alcohol use disorder (AUD), alcohol-related liver disease (ARLD), psychosocial interventions, reduction, abstinence

## Abstract

This systematic review (PROSPERO CRD42021234598) fills a gap in the literature by assessing the efficacy of psychosocial interventions in patients with alcohol use disorder and alcohol-related liver disease (ARLD), focusing on drinking reduction and abstinence as intervention goals. A systematic search for randomized controlled trials (RCTs) was conducted across various databases. Study screening and data extraction were conducted independently by two reviewers. The data were presented through narrative synthesis. Primary outcomes were alcohol reduction and abstinence at the longest follow-up. Ten RCTs were included, evaluating interventions such as cognitive behavioral therapy (CBT), motivational enhancement therapy (MET), motivational interviewing, or peer support. The total population included 1519 participants. Four studies included a combination of more than one intervention, and two trialed an integrated approach, including medical and psychosocial management. A significant reduction was observed with MET, while abstinence was observed with peer support, MET, and CBT/MET within integrated treatment. The overall certainty of the evidence was moderate. Six studies presented a low risk of bias, one had some concerns, and three were high risk. The findings highlight the potential of psychosocial interventions, with MET being repeatedly associated with improved outcomes. Integrated treatment also demonstrated a promising role in ARLD. Future research should head toward improving the robustness and quality of the evidence. It should also aim to further tailor and trial new psychosocial interventions on this specific clinical population. This will enhance the translation of the evidence into real-world settings.

## Introduction

An interaction between environmental, psychosocial, and genetic factors underlies alcohol use disorder (AUD), and subsequent predisposition to alcohol-related liver disease (ARLD) (Stickel et al. 2017)**.** Patients diagnosed with ARLD and AUD face a higher risk of psychological distress, adverse health outcomes, financial hardship and homelessness, and mortality compared to the general population (Johnson et al. 2022). In 2020, 77.8% of alcohol-specific deaths in the UK were attributed to ARLD (Office for National Statistics 2021).

Alcohol abstinence is the therapeutic hallmark to prevent further liver injury (National Institute for Health and Care Excellence 2011; British Liver Trust 2022). Nevertheless, effective interventions to reduce alcohol harm in patients presenting ARLD remain a clinically unmet need, as patients are often excluded from interventional studies due to their physical ill health, alcohol-related brain damage, and different pharmacological profiles. Hence, the lack of effective interventions for comorbid AUD and ARLD is linked to the lack of representative, real-world samples in the existing literature, and it is noted as a research gap (National Institute for Health and Care Excellence 2011). In addition, this population often does not respond well to pharmacological treatment due to hepatoxicity and medication overburden (Subramaniyan et al. 2021). Another obstacle to pharmacological treatment is poor adherence to medications, such as acamprosate, posing a further barrier to the effective clinical management of AUD (Weiss 2004; Zweben et al. 2008).

Psychosocial interventions represent an opportunity to address this treatment gap and are widely recommended for the management of substance misuse and AUD (National Institute for Health and Care Excellence 2011). The provision of brief interventions for behavior change in primary health-care settings has also been effective in reducing harmful alcohol consumption (O’Donnell et al. 2014).

A previous systematic review evaluated the efficacy of psychosocial interventions in maintaining abstinence among patients with ARLD (Khan et al. 2016). Although the data suggested that an integrated approach combining psychosocial interventions with medical care reduced relapse to drinking, the authors also pointed out that no psychosocial intervention was significantly able to maintain abstinence. While the review focused on abstinence as a treatment goal, a gradual approach to abstinence, elicited by reducing drinking, may also benefit this clinical population, especially from an AUD clinical standpoint. Hence, the rationale for the present systematic review is to address this gap in the literature. The present review focuses on the efficacy of psychosocial interventions in reducing alcohol-related harm among patients presenting ARLD and AUD, measured through both reduction and abstinence outcomes.

## Materials and Methods

This systematic review complies with the guidelines established by the Preferred Reporting Items for Systematic reviews and Meta-Analyses (PRISMA) 2020 statement (Page et al. 2021) and the protocol has been registered (PROSPERO: CRD42021234598) (Hemrage et al. 2021).

### Eligibility criteria

#### Study design

The review included randomized controlled trials (RCTs) due to their methodological approach and validity, which assessed the efficacy of psychosocial interventions for the reduction of alcohol consumption among patients presenting ARLD (Sibbald and Roland 1998). Studies published in any language until 31 January 2021 were included. No restrictions were applied on the length of follow-up or setting.

#### Participants

Participants had to be at least 18 years old, with no gender restrictions. Pregnant women were not eligible. A diagnosis of liver disease, presented as alcoholic fatty liver, hepatitis, fibrosis, sclerosis, cirrhosis, or hepatic failure (World Health Organization 2021), was required. Methods of diagnosis include liver biopsy, non-invasive tests, or a clinician diagnosis.

To be included in this systematic review, studies required a minimum of 80% of their samples to have a validated diagnosis AUD, through assessment tools, including clinical interviews, or an AUDIT score >16.

#### Interventions and comparators

Eligible psychosocial interventions recommended for the management of AUD, such as motivational enhancement therapy (MET), motivational interviewing (MI), 12-step facilitation, cognitive behavioral therapy (CBT), behavioral therapy, social network and environment-based therapy, couples therapy, contingency management, counseling, short-term psychodynamic therapy, self-help based interventions, and psychoeducation were included (National Institute for Health and Care Excellence 2011). Interventions could combine elements from across interventions, with no restrictions with regard to duration, modality (individual or group; integrated care), or delivery (inpatient, outpatient, or remotely). Standard care (SC) or any other active interventions were considered as comparators, as defined by the authors in each of the studies.

#### Outcomes

Outcomes of interest included alcohol abstinence and reduction in alcohol consumption, as reported in each of the studies (biologically validated or self-reported), at the longest-follow up available.

### Search strategy and information sources

A comprehensive search strategy following a PICO-style approach and including free text, subject heading, and citation searching to maximize sensitivity and precision was developed. Validated search filters were also integrated into the search strategy (Higgins et al. 2021; NUS Medical Library 2021). The search strategy was tested against a sample of relevant studies and was subject to further refinement. The final search syntax (Supplementary Materials 1) was reviewed by an external senior librarian.

The search was conducted in May 2023 in the following databases: CENTRAL, CINAHL, EMBASE, MEDLINE, PubMed, PsycINFO, Scopus, and Web of Science. Additional literature was searched through citation search and reference lists of RCTs included in the review and additional relevant publications. In the case of missing data or unavailability of full text, study authors were contacted.

Clinical trial and study registers, including the ISRCTN, US National Institutes of Health Ongoing Trials Register, and World Health Organization International Clinical Trials Registry Platform, were also consulted for records published until May 2023 (Supplementary Materials 2).

### Study selection

The search output was deduplicated using Mendeley 1.19.8 (Elsevier 2013) and uploaded to Covidence (Covidence 2014). Title and abstract screening were carried out by the first reviewer (S.H.) and checked by the second reviewer (E.B.). At the full-text level, records were screened independently by the two reviewers, with consultation of third (P.D.) and fourth (C.D.) reviewers in case of disagreement. The eligibility of studies published in languages other than English was confirmed using DeepL, an artificial intelligence translation platform (DeepL GmbH 2018).

### Data collection process and data items

A data extraction form was developed and checked against a sample of relevant papers. Data were independently extracted by the first reviewer (S.H.) and were checked by the second reviewer (E.B.).

### Risk of bias and certainty assessment

Cochrane’s risk of bias 2 (RoB2) tool was used to determine the risk of bias (Sterne et al. 2019). The overall certainty of the evidence of the outcomes was determined using the Grading of Recommendation, Assessment, Development, and Evaluation tool (GRADE) (Guyatt et al. 2008).

### Synthesis methods

Due to clinical and methodological heterogeneity across the studies, a meta-analysis was not conducted. The findings were described narratively. The narrative synthesis was structured by outcomes and informed by the risk of bias and certainty of evidence assessments.

## Results

### Study selection

**Figure 1:**
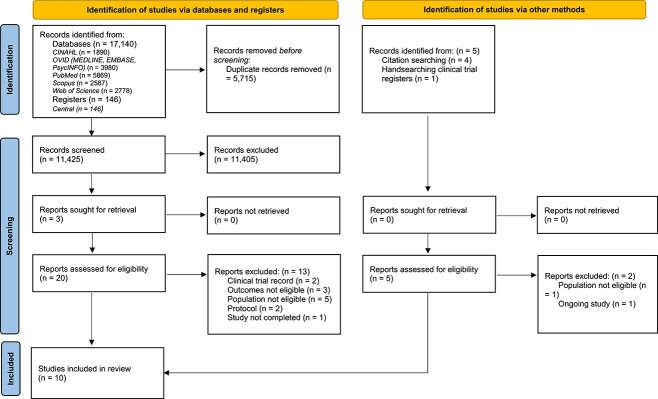
PRISMA flow diagram

The searches identified 17 140 records through database and register searching, and four further studies were identified through citation searching (Fig. 1); one ongoing study was identified by hand-searching clinical trial registers (DiClemente et al. 2021). After the removal of duplicates (*n* = 5715), 11 425 studies were screened at the title and abstract level, and 25 full-text records were independently assessed for eligibility. Fifteen publications were excluded at full-text screening, with main reasons being not including the population or measuring outcomes of interest for our review. (Supplementary Table 1). Ten RCTs were included (Kuchipudi et al. 1990; Willenbring and Olson 1999; Zule et al. 2009; Weinrieb et al. 2011; Dieperink et al. 2014; Shen et al. 2017; DeMartini et al. 2018; Reid et al. 2019;Proeschold-Bell et al. 2020; Stein et al. 2020) of which 4 (Kuchipudi et al. 1990; Willenbring and Olson 1999; Weinrieb et al. 2011; Dieperink et al. 2014) overlap with a previous systematic review on a similar topic (Khan et al. 2016). One of the included studies was published in mandarin and its data were extracted based on the translation process earlier described (Shen et al. 2017; DeepL GmbH 2018).

### Study characteristics

The included RCTs (Kuchipudi et al. 1990; Willenbring and Olson 1999; Zule et al. 2009; Weinrieb et al. 2011; Dieperink et al. 2014; Shen et al. 2017; DeMartini et al. 2018; Reid et al. 2019; Proeschold-Bell et al. 2020; Stein et al. 2020) were carried between 1990 (Kuchipudi et al. 1990) and 2020 (Proeschold-Bell et al. 2020; Stein et al. 2020). Eight trials were conducted in the USA (Kuchipudi et al. 1990; Willenbring and Olson 1999; Zule et al. 2009; Weinrieb et al. 2011; Dieperink et al. 2014; DeMartini et al. 2018; Proeschold-Bell et al. 2020; Stein et al., 2020), one in Australia (Reid et al. 2019), and one in China (Shen et al. 2017) (Supplementary Table 2).

Two RCTs were conducted within Veterans Affair Healthcare services (Willenbring and Olson 1999; Dieperink et al. 2014), three were university affiliated (Weinrieb et al. 2011; DeMartini et al. 2018; Proeschold-Bell et al. 2020), and one was a community outreach study (Zule et al. 2009). Follow-up length varied between 8 (DeMartini et al. 2018; Reid et al. 2019) and 24 months (Willenbring and Olson 1999; Stein et al. 2020).

The overall population included 1519 participants, with sample sizes ranging between 15 (DeMartini et al. 2018) and 625 (Zule et al. 2009)**.** The average age ranged from 41.2 (Zule et al. 2009) to 55.8 (Dieperink et al. 2014); from 0% (Kuchipudi et al. 1990; Willenbring and Olson 1999) to 28.7% (Proeschold-Bell et al. 2020) were identified as female. Four RCTs presented baseline differences in ethnicity (Proeschold-Bell et al. 2020), age (Willenbring and Olson 1999; Reid et al. 2019), AUD (Zule et al. 2009), and comorbidities (Willenbring and Olson 1999), but these were adopted as covariates during statistical analyses (Willenbring and Olson 1999; Zule et al. 2009; Proeschold-Bell et al. 2020) or were not associated with study outcomes (Reid et al. 2019).

Half of the trials recruited participants diagnosed with hepatitis C (HCV) (Zule et al. 2009; Dieperink et al. 2014;Reid et al. 2019; Proeschold-Bell et al. 2020; Stein et al. 2020). Two of the trials aimed to address alcohol consumption in patients awaiting liver transplantation (Weinrieb et al. 2011; DeMartini et al. 2018).

### Summary of interventions

Four RCTs considered reduction (Weinrieb et al. 2011; Dieperink et al. 2014; Reid et al. 2019; Stein et al., 2020) and six considered abstinence (Kuchipudi et al. 1990; Willenbring and Olson 1999; Zule et al. 2009; Shen et al. 2017;DeMartini et al. 2018; Proeschold-Bell et al. 2020) as their primary outcomes (Supplementary Table 3). Within the context of liver transplantation, abstinence was an intervention goal pretransplantation (DeMartini et al. 2018) and posttransplantation (Weinrieb et al. 2011).

The psychosocial interventions adopted in the RCTs include cognitive behavioral RP (DeMartini et al. 2018), MET (Willenbring and Olson 1999; Weinrieb et al. 2011; Dieperink et al. 2014; Stein et al., 2020), MI (Kuchipudi et al. 1990; Zule et al. 2009;Reid et al. 2019; Proeschold-Bell et al. 2020; Stein et al. 2020), BI/BA (Reid et al. 2019; Proeschold-Bell et al. 2020; Stein et al., 2020), peer support (Weinrieb et al. 2011; Shen et al. 2017), and CBT (Willenbring and Olson 1999). The effect of the interventions was compared to counseling (DeMartini et al. 2018), general health education (Zule et al. 2009; Dieperink et al. 2014; Shen et al. 2017), BA only (Stein et al., 2020), and SC (Kuchipudi et al. 1990; Willenbring and Olson 1999; Weinrieb et al. 2011; Shen et al. 2017;Reid et al. 2019; Proeschold-Bell et al. 2020). Four studies included more than one intervention concomitantly, combining aspects of BI and MI (Reid et al. 2019; Proeschold-Bell et al. 2020), MI and MET (Stein et al., 2020), or CBT and MET (Willenbring and Olson 1999). Two RCTs assessed an integrated approach to treatment, combining psychosocial and standard alcohol treatment (Willenbring and Olson 1999; Proeschold-Bell et al. 2020). Two trials delivered their interventions remotely using mobile phones (DeMartini et al. 2018; Stein et al., 2020).

The number of sessions varied between a single 30-min session delivered opportunistically (Reid et al. 2019) to up to 36 sessions delivered over a period of 6 months (Proeschold-Bell et al. 2020), or provided on an individual basis depending on the needs of each patient (Willenbring and Olson 1999).

The interventions were delivered by a spectrum clinicians and trained staff across the studies. These included nurse practitioners (Willenbring and Olson 1999; Reid et al. 2019), a psychiatric nurse (Kuchipudi et al. 1990), licensed psychologists (Shen et al. 2017; Stein et al., 2020), psychiatrists (DeMartini et al. 2018; Proeschold-Bell et al. 2020), or addiction therapists (Weinrieb et al. 2011; Proeschold-Bell et al. 2020). In one of the studies, a MET intervention was delivered a lay member from the community who received the appropriate training (Zule et al. 2009). Further information about each of the psychosocial interventions delivered can be found in Supplementary Tables 2 and 3.

### Effect of psychosocial interventions on reduction outcomes

Five studies reported the effect of psychosocial interventions to reduce drinking (Weinrieb et al. 2011; Dieperink et al. 2014; Reid et al. 2019; Proeschold-Bell et al. 2020; Stein et al., 2020), of which four had reduction as their primary outcome (Weinrieb et al. 2011; Hester et al. 2013; Dieperink et al. 2014; Stein et al., 2020) (Supplementary Table 4). The included RCTs evaluated interventions such as MET only (Weinrieb et al. 2011; Dieperink et al. 2014) or in combination with MI (Stein et al., 2020), or MI combined with BI as part of a SBIRT intervention (Proeschold-Bell et al. 2020).

A statistically significant effect was observed in one of the studies (Weinrieb et al. 2011), comparing a MET intervention to referral to peer support groups and case management to SC, consisting of referral to outpatient services only. Weinrieb *et al*. observed a significant reduction in number of drinks (*P* = 0.003), drinking days (*P* = 0.004), and drinks per drinking day (*P* = 0.035). A MET intervention was also trialed in Dieperink *et al*., with no effect in drinks per week (*P* = 0.57), heavy drinking days (*P* = 0.16), % CDT (*P* = 0.97), and positive % EtG/EtG (*P* = 0.70). The remaining studies (Reid et al. 2019; Proeschold-Bell et al. 2020; Stein et al., 2020) did not provide sufficient statistical power to confidently assert their efficacy.

### Effect of psychosocial interventions on abstinence outcomes

Abstinence was a primary outcome in six studies (Kuchipudi et al. 1990; Willenbring and Olson 1999; Zule et al. 2009; Shen et al. 2017; DeMartini et al. 2018; Proeschold-Bell et al. 2020) (Supplementary Table 5). The overall effect of the psychosocial interventions on abstinence outcomes was significantly favored with MET only (*P* = 0.024) (Dieperink et al. 2014), peer support [Alcoholics Anonymous (AA)] (*P* < 0.05) (Shen et al. 2017), and an integrated approach of CBT combined with MET (*P* = 0.02) (Willenbring and Olson 1999). No statistically significant differences were observed between cognitive behavioral RP (DeMartini et al. 2018), MI only (Kuchipudi et al. 1990; Zule et al. 2009), or combined with BI (Reid et al. 2019; Proeschold-Bell et al. 2020) and their comparators.

### Risk of bias assessment

The RoB 2 assessment classified six studies with low risk of bias regarding constructs, such as their randomization, deviations to intended interventions, missing outcome data, measurement of their outcomes, and selective reporting (Zule et al. 2009; Weinrieb et al. 2011; Dieperink et al. 2014; Shen et al. 2017; DeMartini et al. 2018; Stein et al., 2020). One presented some concerns due to deviations from intended interventions (Willenbring and Olson 1999) and three were high risk of bias (Kuchipudi et al. 1990; Reid et al. 2019; Proeschold-Bell et al. 2020) (Supplementary Figs 1 and 2). Bias due to selective reporting and deviations from the data analysis plan was identified by one study (Reid et al. 2019).

### Certainty of evidence assessment for reduction and abstinence outcomes

The overall level of certainty of evidence, assessed using the GRADE tool (Guyatt et al. 2008), for both reduction and abstinence was moderate (Supplementary Tables 6 and 7). The evidence summarized on reduction outcomes was subject to methodological limitations and imprecision. Borderline concerns were noted on indirectness and inconsistency. At the methodological level, two of the included studies (Reid et al. 2019; Proeschold-Bell et al. 2020) presented a high risk of bias. Additionally, four (Weinrieb et al. 2011; Dieperink et al. 2014; Reid et al. 2019; Stein et al., 2020) RCTs did not detect a significant effect of the intervention and presented large confidence intervals, making the results susceptible to imprecision. As a result, the overall certainty for reduction outcomes was downgraded from high to moderate.

The evidence reviewed on abstinence outcomes was also of moderate certainty. No serious concerns related to indirectness, inconsistency, and publication bias were raised. Three RCTs (Kuchipudi et al. 1990; Reid et al. 2019; Proeschold-Bell et al. 2020) were at high risk of bias, and one presented some concerns (Willenbring and Olson 1999), flagging serious concerns due to methodological limitations. The evidence was also subject to imprecision for their confidence intervals and small sample sizes. This downgraded the overall certainty from high to moderate.

## Discussion

The provision of effective interventions to reduce the harm caused by comorbid AUD and ARLD remains suboptimal. This systematic review aimed to summarize the evidence of the efficacy of psychosocial interventions to address this continued need by looking at reduction and abstinence as treatment goals, adding clinical breadth and depth to the existing literature. The findings highlight the potential of psychosocial intervention for the management of AUD and ARLD, with MET being associated with improved abstinence and reduction outcomes. Integrated treatment also demonstrated a promising role. The existing data on the efficacy of interventions are of moderate certainty; the evidence would benefit from stronger statistical power to further support their efficacy for both reducing drinking and inducing abstinence.

One study comparing MET to SC saw a significant improvement in reduction outcomes (Weinrieb et al. 2011). The authors also noted a link between psychosocial health and the risk of drinking following randomization. Although the MET group showed a significant decrease in drinking, the overall small sample size treatment adherence in both the intervention and control groups should be noted. The RoB2 assessment indicated that Proeschold-Bell *et al*. and Reid *et al*. presented a high risk of bias (Reid et al. 2019; Proeschold-Bell et al. 2020), represented a source of methodological limitations, which, together with outcome indirectness, imprecision, and inconsistency, resulted in a downgrade of certainty of the evidence to moderate. A moderate certainty of the evidence indicated that the true effect is probably close to the estimated effect, but future research should encompass larger samples, narrow confidence intervals, and overall stronger statistical power.

Significant abstinence outcomes were observed with MET, peer support, and integrated outpatient treatment (Willenbring and Olson 1999; Dieperink et al. 2014; Shen et al. 2017). Dieperink *et al*. observed a significant increase in the percentage of days abstinent with a moderate effect size of the intervention with MET, although a significant difference between the control and intervention group was not observed over a 30-day period (Dieperink et al. 2014). The provision of health education sessions to the control group, which may have diluted the effect of the intervention, as well as a short follow-up (3 months), could underly the absence of statistically significant differences. Referral to an AA group intervention significantly decreased drinking and relapse rates, indicating the efficacy of peer support to improve drinking self-management in ARLD (Shen et al. 2017). Cautiously, the authors noted the short-term effect, suggesting longer follow-up periods are needed to further assess the role of the intervention. A third RCT, exploring an integrated outpatient treatment blending components of CBT and MET, saw a significant increase in treatment engagement and abstinence (Willenbring and Olson 1999). The study was the first to evaluate the role of comprehensive, integrated outpatient treatment in ARLD, and evidence showed that this approach significantly engages patients in attending medical appointments beyond alcohol services, serving a clinical population historically unreceptive to treatment or ambivalent to address their alcohol consumption (Grant et al. 2017; Crabb et al. 2020; Madhombiro et al. 2020). Nevertheless, age baseline differences and protocol deviations raised some bias concerns.

The data from the included studies reporting on abstinence outcomes were subject to methodological limitations; these include studies with a high risk of bias (Kuchipudi et al. 1990; Reid et al. 2019; Proeschold-Bell et al. 2020), indirectness from indirect comparisons, imprecision due to diluted intervention effects, as well as inconsistency due to clinical and methodological heterogeneity between studies. Therefore, the similarity between the true and estimated effects is of moderate certainty.

Our review provides novelty to previous findings (Khan et al. 2016). This was attained by applying a wider search and time interval and inclusion of reduction as an outcome of interest, relevant from an addiction treatment clinical standpoint. Four RCTs, trialing CBT, MET, and MI (Kuchipudi et al. 1990; Willenbring and Olson 1999; Weinrieb et al. 2011; Dieperink et al. 2014), overlap between the two reviews; one RCT (Drumright et al. 2011) was not included in our review, as the included participants did not meet the inclusion criteria (see Supplementary table 1). In accordance with the previous review, our review also supports that an integrated, multidisciplinary approach to ARLD can meet the complex needs of patients with comorbid AUD and ARLD. As a continuum, integrated treatment can be either coordinated, with information being shared across providers, or collocated, in which hepatology and addiction services are located in the same setting (Proeschold-Bell et al. 2020; Johnson et al. 2022). In a clinical population that often is unwilling to engage with or faces barriers to treatment, integrated care can address a gap in treatment, particularly for patients who return to medical appointments but are ambivalent about attending alcohol treatment. The remote delivery of interventions might also enable higher adherence and longer follow-ups and address some of the challenges that patients can face with adhering to treatment. An SMS-based relapse prevention intervention from one of the RCTs was interpreted as feasible and acceptable to reach patients beyond a fixed clinical location, favoring health outcomes in a population with poor compliance (Wei et al. 2011; DeMartini et al. 2018). The delivery of psychosocial interventions using mobile technology represents an opportunity for person-centered, real-time support in the patient’s natural environment, which is convenient for disease management (Nesvåg and McKay 2018; Bonfiglio et al. 2022). This allows for ecological momentary interventions to be delivered and continuous support when the patient cannot attend services, such as in the case of physical ill-health or a pandemic. Nonetheless, compound and sequential digital exclusion has been linked to socio-economically disadvantaged groups. This can hinder the access to information and communication technologies, impacting on the likelihood of a patient to be actively engaged with interventions or services (Martin et al. 2016; Van Deursen et al. 2017).

Our review presents several strengths. Our findings add clinical depth and breadth to the current knowledge on ARLD treatment by being the first systematic review to account for a stepped approach to abstinence. Our search was comprehensive, relying on various databases and did not include restrictions on language, widening the scope, and novelty of our review. We restricted the study design to RCTs, given their methodological approach, granting them the highest validity (Sibbald and Roland 1998). The review presents a few limitations, such as the clinical and methodological heterogeneity across the studies. As a result, it was not possible to conduct a meta-analysis to draw direct comparisons between the studies. Some concerns were raised with selective reporting or detection bias, which downgraded the risk of bias assessment in some of the RCTs (Kuchipudi et al. 1990; Willenbring and Olson 1999; Reid et al. 2019; Proeschold-Bell et al. 2020). Blinding of participants or staff delivering interventions was not always feasible due to study design; this increased the susceptibility of domains such as assignment to intervention or outcome measurement to bias (Dieperink et al. 2014; Proeschold-Bell et al. 2020). As such, the overall findings were of moderate certainty. Nevertheless, they assert the promising scope of psychosocial interventions to reduce harm in ARLD, directing future research toward the inclusion of larger samples and follow-up times. Patients in this clinical group would also benefit from psychosocial interventions tailored to their needs.

## Conclusion

In summary, there is a promising scope for the efficacy of psychosocial interventions to reduce harm in ARLD, in which an integrated treatment can relieve barriers that this clinical population faces when engaging with services. MET was also associated with improved outcomes. Studies trialing new psychosocial interventions, accounting for the complex needs of this clinical population, should be at the core of future research.

## Authors’ contributions

S.H.: first reviewer; conceptualization, protocol design, methodology, data screening, data extraction formal analysis, writing - original draft, review and editing; E.B.: second reviewer; screening and data extraction, writing - review and editing; P.D.: third reviewer; conceptualization, protocol revision, writing - review and editing; C.D.: fourth reviewer; conceptualization, protocol revision, writing - review and editing.

## Conflict of interest

None declared.

## Funding

This work was supported by the National Institute for Health Research (NIHR) Applied Research Collaboration (ARC) South London (grant number NIHR200152). The views expressed are those of the authors and not necessarily those of the, the NIHR, or the Department of Health and Social Care. C.D. and P.D. were supported by the NIHR Specialist Biomedical Research Centre for Mental Health at South London and Maudsley NHS Foundation Trust, King’s College London. They were also supported by the NIHR Collaboration for Leadership in Applied Health Research and Care at King’s College Hospital NHS Foundation Trust and the National Institute for Health Research Applied Research Collaboration South London (NIHR ARC South London) at King’s College Hospital NHS Foundation Trust. C.D. was supported by an NIHR Senior Investigator Award.

## Data availability

The data underlying this article are available in the article and in its online supplementary material.

## Supplementary Material

Supplementary_material_agad051
