# Supplementary material for: Efficacy of psychosocial interventions to reduce alcohol use in comorbid alcohol use disorder and alcohol-related liver disease: a systematic review of randomized controlled trials
Source: Alcohol Alcohol. 2023 Aug 1;58(5):478–84. doi: 10.1093/alcalc/agad051 (PMC10493519; doi:10.1093/alcalc/agad051)
Supplement: Supplementary_material_agad051 [file supplementary_material_agad051.docx]

**Supplementary Materials 1: Database search strategies**

**#1 = Alcoholic liver disease**

**#2 = Psychosocial Interventions**

**#3 = RCT filter**

**Ovid databases (Medline, EMBASE, PsycINFO)**

**#1**

1. alcohol* fatty liver.mp.
2. exp Fatty Liver, Alcoholic/
3. exp Hepatitis, Alcoholic/
4. alcohol* hepatitis.mp.
5. alcohol* fibrosis.mp.
6. alcohol* sclerosis.mp.
7. alcohol* cirrhosis.mp.
8. exp Liver Cirrhosis, Alcoholic/
9. alcohol* hepatic failure.mp.
10. alcohol-related disorders/
11. alcohol-induced disorders/
12. alcoholic intoxication/
13. alcoholism/ or binge drinking/
14. alcohol-related disorders.mp. or exp Alcohol-Related Disorders/
15. alcohol-induced disorders.mp. or exp Alcohol-Induced Disorders/
16. exp Liver Diseases/ or liver diseases.mp.
17. (liver disease* or cirrho* or (liver and fibrosis) or steatosis or hepatic failure or liver failure).mp.
18. alcoholic liver disease.mp. or exp Liver Diseases, Alcoholic/
19. liver diseases, alcoholic/ or fatty liver, alcoholic/ or hepatitis, alcoholic/ or liver cirrhosis, alcoholic/
20. 1 or 2 or 3 or 4 or 5 or 6 or 7 or 8 or 9 or 10 or 11 or 12 or 13

**#2**

1. psychosocial intervention.mp.
2. (motivational enhancement therapy or "MET").mp.
3. exp Behavior Therapy/ or behavio* therapy.mp.
4. motivational interview*.mp. or exp Motivational Interviewing/
5. exp Self-Help Groups/
6. (twelve step facilitation or twelve step program or TSF).mp.
7. behavior control/ or community mental health services/ or counselling/ or directive counselling/ or distance counselling/ or "acceptance and commitment therapy"/ or mindfulness/ or feedback, psychological/
8. (cognitive behavio?ral therapy or "CBT").mp. or exp Cognitive Behavio?ral Therapy/
9. (social network environmental therapy or "SBNT").mp.
10. couples therapy/ or family therapy/
11. exp Psychotherapy, Psychodynamic/ or exp Psychotherapy, Brief/ or exp Psychotherapy, Rational-Emotive/ or psychotherapy.mp. or exp Psychotherapy/ or exp Psychotherapy, Multiple/ or exp Psychotherapy, Group/ or exp Imagery, Psychotherapy/ or exp Person-Centred Psychotherapy/
12. counselling.mp. or exp Counselling/
13. exp Patient Education as Topic/ or psychoeducation.mp.
14. sensitivity training group.mp. or exp Sensitivity Training Groups/
15. residential treatment.mp. or exp Residential Treatment/
16. substance abuse treatment centres.mp. or exp Substance Abuse Treatment Centres/
17. exp "Delivery of Health Care, Integrated"/ or integrated care.mp.
18. alcoholism/rhc
19. 1 or 2 or 3 or 4 or 5 or 6 or 7 or 8 or 9 or 10 or 11 or 12 or 13 or 14 or 15 or 16 or 17 or 18

**#3 (Cochrane RCT research filter)**

1. randomised controlled trial.pt.
2. controlled clinical trial.pt.
3. randomised.ab.
4. placebo.ab.
5. clinical trials as topic.sh.
6. randomly.ab.
7. trial.ti.
8. 1 or 2 or 3 or 4 or 5 or 6 or 7
9. exp animals/ not humans.sh.
10. 8 not 9

**#1 AND #2 AND #3**

**Scopus**

**#1**

( TITLE-ABS-KEY ( "alcohol* fatty liver" ) )  OR  ( INDEXTERMS ( "Fatty Liver, Alcoholic" ) )  OR  ( INDEXTERMS ( "Hepatitis, Alcoholic" ) )  OR  ( TITLE-ABS-KEY ( "alcohol* hepatitis" ) )  OR  ( TITLE-ABS-KEY ( "alcohol* fibrosis" ) )  OR  ( TITLE-ABS-KEY ( "alcohol* sclerosis" ) )  OR  ( TITLE-ABS-KEY ( "alcohol* cirrhosis" ) )  OR  ( INDEXTERMS ( "Liver Cirrhosis, Alcoholic" ) )  OR  ( TITLE-ABS-KEY ( "alcohol* hepatic failure" ) )  OR  ( INDEXTERMS ( "alcohol-related disorders" ) )  OR  ( INDEXTERMS ( "alcohol-induced disorders" ) )  OR  ( INDEXTERMS ( "alcoholic intoxication" ) )  OR  ( INDEXTERMS ( "alcoholism" )  OR  INDEXTERMS ( "binge drinking" ) )  OR  ( TITLE-ABS-KEY ( "alcohol-related disorders" )  OR  INDEXTERMS ( "Alcohol-Related Disorders" ) )  OR  ( TITLE-ABS-KEY ( "alcohol-induced disorders" )  OR  INDEXTERMS ( "Alcohol-Induced Disorders" ) )  OR  ( INDEXTERMS ( "Liver Diseases" )  OR  TITLE-ABS-KEY ( "liver diseases" ) )  OR  ( ( TITLE-ABS-KEY ( "liver disease*" )  OR  TITLE-ABS-KEY ( "cirrho*" )  OR  ( TITLE-ABS-KEY ( "liver" )  AND  TITLE-ABS-KEY ( "fibrosis" ) )  OR  TITLE-ABS-KEY ( "steatosis" )  OR  TITLE-ABS-KEY ( "hepatic failure" )  OR  TITLE-ABS-KEY ( "liver failure" ) ) )  OR  ( TITLE-ABS-KEY ( "alcoholic liver disease" )  OR  INDEXTERMS ( "Liver Diseases, Alcoholic" ) )  OR  ( INDEXTERMS ( "liver diseases, alcoholic" )  OR  INDEXTERMS ( "fatty liver, alcoholic" )  OR  INDEXTERMS ( "hepatitis, alcoholic" )  OR  INDEXTERMS ( "liver cirrhosis, alcoholic" ) )

**#2**

( ( TITLE-ABS-KEY ( "psychosocial intervention" ) )  OR  ( ( TITLE-ABS-KEY ( "motivational enhancement therapy" )  OR  TITLE-ABS-KEY ( "MET" ) ) )  OR  ( INDEXTERMS ( "Behavior Therapy" )  OR  TITLE-ABS-KEY ( "behavio* therapy" ) )  OR  ( TITLE-ABS-KEY ( "motivational interview*" )  OR  INDEXTERMS ( "Motivational Interviewing" ) )  OR  ( INDEXTERMS ( "Self-Help Groups" ) )  OR  ( ( TITLE-ABS-KEY ( "twelve step facilitation" )  OR  TITLE-ABS-KEY ( "twelve step program" )  OR  TITLE-ABS-KEY ( "TSF" ) ) )  OR  ( INDEXTERMS ( "behavior control" )  OR  INDEXTERMS ( "community mental health services" )  OR  INDEXTERMS ( "counselling" )  OR  INDEXTERMS ( "directive counselling" )  OR  INDEXTERMS ( "distance counselling" )  OR  INDEXTERMS ( "acceptance and commitment therapy" )  OR  INDEXTERMS ( "mindfulness" )  OR  INDEXTERMS ( "feedback, psychological" ) )  OR  ( ( TITLE-ABS-KEY ( "cognitive behavio?ral therapy" )  OR  TITLE-ABS-KEY ( "CBT" ) )  OR  INDEXTERMS ( "Cognitive Behavio?ral Therapy" ) )  OR  ( ( TITLE-ABS-KEY ( "social network environmental therapy" )  OR  TITLE-ABS-KEY ( "SBNT" ) ) )  OR  ( INDEXTERMS ( "couples therapy" )  OR  INDEXTERMS ( "family therapy" ) )  OR  ( INDEXTERMS ( "Psychotherapy, Psychodynamic" )  OR  INDEXTERMS ( "Psychotherapy, Brief" )  OR  INDEXTERMS ( "Psychotherapy, Rational-Emotive" )  OR  TITLE-ABS-KEY ( "psychotherapy" )  OR  INDEXTERMS ( "Psychotherapy" )  OR  INDEXTERMS ( "Psychotherapy, Multiple" )  OR  INDEXTERMS ( "Psychotherapy, Group" )  OR  INDEXTERMS ( "Imagery, Psychotherapy" )  OR  INDEXTERMS ( "Person-Centred Psychotherapy" ) )  OR  ( TITLE-ABS-KEY ( "counselling" )  OR  INDEXTERMS ( "Counselling" ) )  OR  ( INDEXTERMS ( "Patient Education as Topic" )  OR  TITLE-ABS-KEY ( "psychoeducation" ) )  OR  ( TITLE-ABS-KEY ( "sensitivity training group" )  OR  INDEXTERMS ( "Sensitivity Training Groups" ) )  OR  ( TITLE-ABS-KEY ( "residential treatment" )  OR  INDEXTERMS ( "Residential Treatment" ) )  OR  ( TITLE-ABS-KEY ( "substance abuse treatment centres" )  OR  INDEXTERMS ( "Substance Abuse Treatment Centres" ) )  OR  ( INDEXTERMS ( "Delivery of Health Care, Integrated" )  OR  TITLE-ABS-KEY ( "integrated care" ) )  OR  ( INDEXTERMS ( "alcoholism" ) ) )

**#3** **(NUS Medical RCT search filter)**

( INDEXTERMS ( "clinical trials"  OR  "clinical trials as a topic"  OR  "randomised controlled trial"  OR  "Randomised Controlled Trials as Topic"  OR  "controlled clinical trial"  OR  "Controlled Clinical Trials"  OR  "random allocation"  OR  "Double-Blind Method"  OR  "Single-Blind Method"  OR  "Cross-Over Studies"  OR  "Placebos"  OR  "multicenter study"  OR  "double blind procedure"  OR  "single blind procedure"  OR  "crossover procedure"  OR  "clinical trial"  OR  "controlled study"  OR  "randomization"  OR  "placebo" ) )  OR  ( TITLE-ABS-KEY ( ( "clinical trials"  OR  "clinical trials as a topic"  OR  "randomised controlled trial"  OR  "Randomised Controlled Trials as Topic"  OR  "controlled clinical trial"  OR  "Controlled Clinical Trials as Topic"  OR  "random allocation"  OR  "randomly allocated"  OR  "allocated randomly"  OR  "Double-Blind Method"  OR  "Single-Blind Method"  OR  "Cross-Over Studies"  OR  "Placebos"  OR  "cross-over trial"  OR  "single blind"  OR  "double blind"  OR  "factorial design"  OR  "factorial trial" ) ) )  OR  ( TITLE-ABS ( clinical  AND trial*  OR  trial*  OR  rct*  OR  random*  OR  blind* ) )

**#1 AND #2 AND #3**

**CINAHL (via EBSCO Host)**

**S1** ("alcohol* fatty liver") OR ((MH "Fatty Liver, Alcoholic+")) OR ((MH "Hepatitis, Alcoholic+")) OR ("alcohol* hepatitis") OR ("alcohol* fibrosis") OR ("alcohol* sclerosis") OR ("alcohol* cirrhosis") OR ((MH "Liver Cirrhosis, Alcoholic+")) OR ("alcohol* hepatic failure") OR ((MH "alcohol-related disorders")) OR ((MH "alcohol-induced disorders")) OR ((MH "alcoholic intoxication")) OR ((MH "alcoholism") OR (MH "binge drinking")) OR ("alcohol-related disorders" OR (MH "Alcohol-Related Disorders+")) OR ("alcohol-induced disorders" OR (MH "Alcohol-Induced Disorders+")) OR ((MH "Liver Diseases+") OR "liver diseases") OR (("liver disease*" OR cirrho* OR (liver AND fibrosis) OR steatosis OR "hepatic failure" OR "liver failure")) OR ("alcoholic liver disease" OR (MH "Liver Diseases, Alcoholic+")) OR ((MH "liver diseases, alcoholic") OR (MH "fatty liver, alcoholic") OR (MH "hepatitis, alcoholic") OR (MH "liver cirrhosis, alcoholic"))

**S2** ("psychosocial intervention") OR (("motivational enhancement therapy" OR MET)) OR ((MH "Behavior Therapy+") OR "behavio* therapy") OR ("motivational interview*" OR (MH "Motivational Interviewing+")) OR ((MH "Self-Help Groups+")) OR (("twelve step facilitation" OR "twelve step program" OR TSF)) OR ((MH "behavior control") OR (MH "community mental health services") OR (MH "counselling") OR (MH "directive counselling") OR (MH "distance counselling") OR (MH "acceptance and commitment therapy") OR (MH "mindfulness") OR (MH "feedback, psychological")) OR (("cognitive behavio#ral therapy" OR CBT) OR (MH "Cognitive Behavio?ral Therapy+")) OR (("social network environmental therapy" OR SBNT)) OR ((MH "couples therapy") OR (MH "family therapy")) OR ((MH "Psychotherapy, Psychodynamic+") OR (MH "Psychotherapy, Brief+") OR (MH "Psychotherapy, Rational-Emotive+") OR psychotherapy OR (MH "Psychotherapy+") OR (MH "Psychotherapy, Multiple+") OR (MH "Psychotherapy, Group+") OR (MH "Imagery, Psychotherapy+") OR (MH "Person-Centred Psychotherapy+")) OR (counselling OR (MH "Counselling+")) OR ((MH "Patient Education as Topic+") OR psychoeducation) OR ("sensitivity training group" OR (MH "Sensitivity Training Groups+")) OR ("residential treatment" OR (MH "Residential Treatment+")) OR ("substance abuse treatment centres" OR (MH "Substance Abuse Treatment Centres+")) OR ((MH "Delivery of Health Care, Integrated+") OR "integrated care") OR ((MH "alcoholism"))

**S3** **(Cochrane RCT research filter)** ((PT "randomised controlled trial") OR (PT "controlled clinical trial") OR (AB randomised) OR (AB placebo) OR ((MH "clinical trials as topic")) OR (AB randomly) OR (TI trial)) NOT ((MH "animals+") NOT (MH "humans"))

**S1 AND S2 AND S3**

**PubMed**

**#1**

"fatty liver, alcoholic"[MeSH Terms] OR "hepatitis, alcoholic"[MeSH Terms] OR "liver cirrhosis, alcoholic"[MeSH Terms] OR "alcohol related disorders"[MeSH Terms:noexp] OR "alcohol induced disorders"[MeSH Terms:noexp] OR "alcoholic intoxication"[MeSH Terms:noexp] OR ("alcoholism"[MeSH Terms:noexp] OR "binge drinking"[MeSH Terms:noexp]) OR ("alcohol related disorders"[Text Word] OR "alcohol related disorders"[MeSH Terms]) OR ("alcohol induced disorders"[Text Word] OR "alcohol induced disorders"[MeSH Terms]) OR ("Liver Diseases"[MeSH Terms] OR "Liver Diseases"[Text Word]) OR ("liver disease*"[Text Word] OR "cirrho*"[Text Word] OR ("liver"[Text Word] AND "fibrosis"[Text Word]) OR "steatosis"[Text Word] OR "hepatic failure"[Text Word] OR "liver failure"[Text Word]) OR ("alcoholic liver disease"[Text Word] OR "liver diseases, alcoholic"[MeSH Terms]) OR ("liver diseases, alcoholic"[MeSH Terms:noexp] OR "fatty liver, alcoholic"[MeSH Terms:noexp] OR "hepatitis, alcoholic"[MeSH Terms:noexp] OR "liver cirrhosis, alcoholic"[MeSH Terms:noexp])

**#2**

"psychosocial intervention"[Text Word] OR "motivational enhancement therapy"[Text Word] OR "MET"[Text Word] OR "Behavior Therapy"[MeSH Terms] OR "motivational interview*"[Text Word] OR "Motivational Interviewing"[MeSH Terms] OR "Self-Help Groups"[MeSH Terms] OR "twelve step facilitation"[Text Word] OR "twelve step program"[Text Word] OR "TSF"[Text Word] OR "behavior control"[MeSH Terms:noexp] OR "community mental health services"[MeSH Terms:noexp] OR "counseling"[MeSH Terms:noexp] OR "acceptance and commitment therapy"[MeSH Terms:noexp] OR "mindfulness"[MeSH Terms:noexp] OR "feedback, psychological"[MeSH Terms:noexp] OR "CBT"[Text Word] OR "SBNT"[Text Word] OR "couples therapy"[MeSH Terms:noexp] OR "family therapy"[MeSH Terms:noexp] OR "psychotherapy, psychodynamic"[MeSH Terms] OR "psychotherapy, brief"[MeSH Terms] OR "psychotherapy, rational emotive"[MeSH Terms] OR "psychotherapy"[Text Word] OR "psychotherapy"[MeSH Terms] OR "psychotherapy, multiple"[MeSH Terms] OR "psychotherapy, group"[MeSH Terms] OR "imagery, psychotherapy"[MeSH Terms] OR "counselling"[Text Word] OR "counseling"[MeSH Terms] OR "Patient Education as Topic"[MeSH Terms] OR "psychoeducation"[Text Word] OR "sensitivity training group"[Text Word] OR "Sensitivity Training Groups"[MeSH Terms] OR "Residential Treatment"[Text Word] OR "Residential Treatment"[MeSH Terms] OR "substance abuse treatment centres"[Text Word] OR "delivery of health care, integrated"[MeSH Terms] OR "integrated care"[Text Word] OR "alcoholism"[MeSH Terms:noexp]

**#3** **(Cochrane RCT research filter)**

("randomised controlled trial"[Publication Type] OR "controlled clinical trial"[Publication Type] OR "randomised"[Title/Abstract] OR "placebo"[Title/Abstract] OR "clinical trials as topic"[MeSH Terms:noexp] OR "randomly"[Title/Abstract] OR "trial"[Title]) NOT ("animals"[MeSH Terms] NOT "humans"[MeSH Terms:noexp])

**#1 AND #2 AND #2**

**CENTRAL**

**#1** ("alcohol* fatty liver":ti,ab,kw) OR ([mh "Fatty Liver, Alcoholic"]) OR ([mh "Hepatitis, Alcoholic"]) OR ("alcohol* hepatitis":ti,ab,kw) OR ("alcohol* fibrosis":ti,ab,kw) OR ("alcohol* sclerosis":ti,ab,kw) OR ("alcohol* cirrhosis":ti,ab,kw) OR ([mh "Liver Cirrhosis, Alcoholic"]) OR ("alcohol* hepatic failure":ti,ab,kw) OR ([mh ^"alcohol-related disorders"]) OR ([mh ^"alcohol-induced disorders"]) OR ([mh ^"alcoholic intoxication"]) OR ([mh ^alcoholism] OR [mh ^"binge drinking"]) OR ("alcohol-related disorders":ti,ab,kw OR [mh "Alcohol-Related Disorders"]) OR ("alcohol-induced disorders":ti,ab,kw OR [mh "Alcohol-Induced Disorders"]) OR ([mh "Liver Diseases"] OR "liver diseases":ti,ab,kw) OR (("liver disease*" OR cirrho* OR (liver AND fibrosis) OR steatosis OR "hepatic failure" OR "liver failure"):ti,ab,kw) OR ("alcoholic liver disease":ti,ab,kw OR [mh "Liver Diseases, Alcoholic"]) OR ([mh ^"liver diseases, alcoholic"] OR [mh ^"fatty liver, alcoholic"] OR [mh ^"hepatitis, alcoholic"] OR [mh ^"liver cirrhosis, alcoholic"])

**#2** "psychosocial intervention”

**#1 AND #2**

**Web of Science**

**#1** TS = (alcohol* fatty liver OR alcohol* hepatitis OR alcohol* fibrosis OR alcohol* sclerosis OR alcohol* cirrhosis OR ("Liver Cirrhosis, Alcoholic") OR alcohol* hepatic failure OR alcohol-related disorders OR alcohol-induced disorders OR alcoholic intoxication OR alcoholism OR binge drinking)

**#2**  TS = (psychosocial intervention OR motivational enhancement therapy OR MET OR Behavior Therapy OR behavio* therapy OR motivational interview* OR Motivational Interviewing OR Self-Help Groups OR twelve step facilitation OR twelve step program OR TSF OR behavior control OR community mental health services OR counselling OR directive counselling OR distance counselling OR acceptance and commitment therapy OR mindfulness OR psychological feedback OR cognitive behavio*ral therapy OR CBT OR Cognitive Behavio*ral Therapy OR social network environmental therapy OR SBNT OR couples therapy OR family therapy OR psychodynamic psychotherapy OR brief psychotherapy OR rational-emotive psychotherapy OR psychotherapy OR Psychotherapy OR multiple psychotherapy OR group psychotherapy OR psychotherapy imagery OR Person-Centred Psychotherapy OR counselling OR Counselling OR Patient Education as Topic OR psychoeducation OR sensitivity training group OR Sensitivity Training Groups OR residential treatment OR Residential Treatment OR substance abuse treatment centres OR Substance Abuse Treatment Centres OR Delivery of Health Care, Integrated OR integrated care OR alcoholism)

**#3** **(Cochrane RCT research filter)** TS=(randomised OR randomised OR randomisation OR randomisation OR placebo* OR (random* AND (allocat* OR assign*) ) OR (blind* AND (single OR double OR treble OR triple) ))

**#1 AND #2 AND #3**

**Supplementary Materials 2: Clinical Trial Registers - search strategies**

**ISRCTN Registry**

(Condition: Alcoholic liver disease AND Interventions: Psychosocial interventions)

**US National Institutes of Health Ongoing Trials Register (ClinicalTrials.gov)**

Condition: Alcoholic Liver Disease

**WHO International Clinical Trials Registry Platform**

Condition: Alcoholic Liver Disease

Intervention: Psychosocial Intervention

**Supplementary Table 1: List of records excluded at full text**

| Study | Reason for exclusion |
| --- | --- |
| Bailey (2017) | Outcomes of interest not measured. |
| DiClemente (2021) | Ongoing study. |
| Dieperink (2012) | Study protocol. |
| Drumright (2011) | Population of interest not included: only 47.9% of the population had an AUDIT score indicated harmful drinking or alcohol dependence |
| Edelman (2019) | Population of interest not included: baseline AUDIT-C scores did not indicate harmful drinking nor alcohol dependence. |
| Fink (2016) | Outcomes of interest not measured. |
| Groessl (2013) | Population of interest not included: baseline AUDIT scores did not indicate harmful drinking nor alcohol dependence. |
| Irvine (2017) | Population of interest not included criteria: no ARLD. |
| NCT00598416 (2008) | Clinical trial record. |
| NCT03402256 (2018) | Clinical trial record. |
| North (2017) | Population of interest not included: only 10% AUD at the time of the study. |
| Proeschold-Bell (2018) | Study protocol. |
| Constant (2021) | Population of interest not included: no ARLD. |
| Sussman (2005) | Outcomes of interest not measured. |
| Weinrieb (2001) | Study interrupted. |

***Legend: List of records found during searches excluded at full text screening and respective reasons for exclusion.***

**Supplementary Table 2: Summary of characteristics of the included RCTs**

|  | | | | | |
| --- | --- | --- | --- | --- | --- |
| **Study** | **Funding and COI** | **Country, setting and data collection period** | **Study design* and longest follow-up** | **Sample demographics and diagnosis** | **Inclusion criteria** |
| DeMartini (2018) | Yale-New Haven Hospital (Department of Psychological Medicine)  SO is a member of the Alcohol Clinical Trial Initiative; BT received a grant from Pfizer for medicine and testified in litigation against tobacco companies; no COI from remaining authors | USA  Yale-New Haven Hospital Transplantation Centre  March 2013 to March 2014 | Randomised pilot feasibility trial with two groups: standard care (SC) only compared to a SMS-based cognitive behavioural relapse prevention (RP) plus SC  8 weeks | Participants: n = 14 (SC only n = 6, RP plus SC n = 8)  Mean age (SD): 50.8 (7.9)  Female, % (n): 27 (4)  No significant demographic differences between groups at baseline  ARLD; awaiting liver transplantation | ≥ 1 drinking episode in the previous year; ARLD diagnosis; evaluation phase for liver transplantation listing |
| Dieperink (2014) | Research Service of the Department of Veterans Affairs (VA)  ED, BF and PH received research funding from the Department of VA; no COI from remaining authors | USA  VA Health Care Systems hepatitis clinics (Minneapolis and Portland)  November 2008 to July 2012 | Randomised, single-blind, controlled trial  with two arms: motivational enhancement therapy (MET) and control health education (EDU)  6 months | Participants: n = 138 (MET n = 70, EDU n = 68)  Mean age (SD): MET 55.8 (6.8) EDU 55.2 (6.3)  Female, % (n): MET 2.9 (2) EDU 5.9 (4)  No significant demographic differences between groups at baseline  HCV and AUD | Confirmed chronic HCV diagnosis; DSM-IV-TR alcohol dependence/abuse diagnosis; veterans aged over 18 years old currently using alcohol: >7 standard drinks per week/at least one heavy drinking (>4 standard drinks) two weeks prior |
| Kuchipudi (1990) | Funding and COI not specified | USA  Acute medical unit  Data collection period not reported | Randomised controlled trial with two arms: motivational interviewing (MI) and SC  16 weeks | Participants: n = 114 (SC n = 55 MI n = 59)  Mean age (SE): SC 53 (16) MI 51 (13)  Female, % (n): 0  No significant demographic differences between groups at baseline  Pancreatitis, alcoholic liver disease, peptic ulcer with gastritis | Diagnosis of pancreatitis, alcoholic liver disease, peptic ulcer with gastritis; currently drinking; received previous medical advice to abstain from drinking |
| Proeschold-Bell (2020) | National Institutes of Health and Duke University Center for AIDS Research  MF, DE, PM, AM and SN provided consultancy and received grants from pharmaceutical companies | USA  Liver centres  October 2014 to September 2018 | Unblinded, randomised, pragmatic trial with two arms: Screening, Brief Intervention, and Referral to Treatment (SBIRT) only (BI with MI) and SBIRT (BI with MI) plus alcohol treatment  12 months | Participants: n = 181 (SBIRT only n = 86, SBIRT plus treatment n = 95)  Mean age (SD): 54.9 (SD 9.5)  Female, % (n): 28.7 (52)  Ethnicity significantly differed across treatment arms (p = 0.003); ethnicity included as covariate during statistical analysis  HCV | Confirmed chronic HCV diagnosis; AUDIT ≥ 4 for women, ≥ 8 for men; at least one alcoholic drink in the previous 60 days; aged 18 or over; ability to understand and speak English; not engaged in substance abuse treatment at the time of the study; informed consent; ability to attend at least one individual therapy session |
| Reid (2019) | Royal Brisbane & Women’s Hospital Foundation Research Grants and Department of Gastroenterology (Royal Brisbane & Women’s Hospital)  No COI | Australia  Outpatient hepatology clinic  June 2013 to December 2014 | Randomised controlled trial with two arms: SC only (active comparator) and brief intervention (BI) plus SC  8 weeks | Participants: n = 65 (SC n = 31 BI n = 34)  Mean age (SD): SC 51 (9.4) BI 45.6 (SD 8.1)  Female: SC 22.6 (7) BI 22.9 (8)  SC group significantly older than BI group (p = 0.015)  HCV | Alcohol consumption; aged over 18 years; ability to read and speak English; attending ambulatory hepatology clinic for management of chronic HCV. |
| Shen (2017) | Funding and COI not specified | China  Outpatient/inpatient department of Gastroenterology  February 2015 to June 2016 | Randomised controlled trial with two groups: SC and peer support (AA)  3 months | Participants: n = 79 (SC n = 39, AA n = 40)  Mean age (SD): 43.78 (11.23)  Female, % (n): 22.7 (18)  No significant demographic differences between groups at baseline  ARLD | Chinese Medical Association ARLD diagnosis; aged between 18 and 70 years old. |
| Stein (2020) | National Institutes of Health  MS is involved in grant revision for research; no COI from remaining authors | USA  Three outpatient HIV clinics  December 2014 to June 2020 | Randomised clinical trial with two groups: brief advice (BA) and MI combined with MET  24 months | Participants: n = 110 (BA = 53, MI/MET = 57)  Mean age (SD): 51.4 (10)  Female, % (n): 18.2 (20)  No significant demographic differences between groups at baseline  HCV | Aged 18 or older; living with HIV/AIDS (PLWHA); confirmed diagnosis of HCV; ≥ 4 alcoholic drinks/week during the previous month; access to a phone |
| Weinrieb (2011) | National Institute on Alcohol Abuse and Alcoholism  COI not specified | USA  Two university-based hospitals  June 2000 to January 2004 | Randomised controlled trial with two arms: SC and MET  96 weeks | Participants: n = 91 (SC n = 45, MET n = 46)  Mean age (range): SC 48.0 (43.0-52.0) MET 50.5 (42.0-56)  Female, % (n): SC 17.8 (8) MET 15.2 (7)  No significant demographic differences between groups at baseline  ARLD, awaiting liver transplantation | Alcohol as primary cause of liver disease; ≥ 1 drink within 2 years prior to initial transplant evaluation; ability to provide informed consent |
| Willenbring (1999) | Department of VA Research Service  COI not specified | USA  Inpatient and outpatient care, Minneapolis Veterans Affair Medical Centre  Data collection period not reported | Randomised controlled trial with two arms: SC and integrated outpatient treatment (IOT)  24 months | Participants: n = 101 (SC n = 53, IOT n = 48)  Mean age (SD): IOT 52.8 (11.5) control 57.2 (10.0)  Female, % (n): 0  IOT group significant younger than control group, indicated more psychological problems and pancreatitis diagnosis (p = 0.01); age included as covariate during statistical analysis  ALRD (cirrhosis or symptomatic alcoholic hepatitis), alcoholic pancreatitis, alcohol-related gastrointestinal bleeding | Diagnosis of severe alcohol-related medical illness (cirrhosis, hepatitis, pancreatitis, cardiomyopathy, gastrointestinal bleeding requiring hospitalization, neuropathy); pathological drinking during previous 6 months; ability to attend monthly clinic visits |
| Zule (2009) | National Institute on Drug Abuse, University of North Carolina Centre for AIDS Research program (National Institutes of Health)  COI not specified | USA  Street/community outreach  2003 to 2006 | Randomised trial with two arms: EDU and MI  12 months | Participants: n = 625  Mean age (SD): 41.2 (9.3)  Female, % (n): 27.1 (169)  Alcohol use significantly higher in MI group (p = 0.04); baseline alcohol use adjusted in multiple logistic regression analyses  HCV | Aged 18 or older; self-reported IDU in previous 30 days; visible injection marks or positive urine test for heroin, cocaine and methamphetamine; no formal substance use treatment in previous 30 days; current residence in one of the two study counties |

*As described by study authors.

AIDS: acquired immunodeficiency syndrome; ARLD: alcohol-related liver disease; AUDIT; BA: brief advice; BI: brief intervention; COI: conflicts of interest; DSM-IV-TR:  Diagnostic and Statistical Manual of Mental Disorders, 4^th^ Edition, Text Revision; EDU: health education; HCV: hepatitis C virus; HIV: human immunodeficiency virus; IDU: injection drug users; IOT: integrated outpatient treatment; MET: motivational enhancement therapy; MI: motivational interviewing; PLWHA: People living with HIV/AIDS; SC: standard care; SD: standard deviation; SE: standard error; TM: text message; USA: United States of America; VA: Veterans Affairs

***Legend: summary of funding, conflicts of interest, country, setting, data collection period, study design, follow-up, demographic information, ARLD diagnosis and inclusion criteria of the RCTs included in this review.***

**Supplementary Table 3: Summary of findings of the included RCTs**

|  | | | | |
| --- | --- | --- | --- | --- |
| **Study** | **Intervention goal*, primary outcome** | **Study conditions (components, sessions, duration, modality, staff, concurrent pharmacotherapy use for alcohol management)** | **Reduction outcomes** | **Abstinence outcomes** |
| DeMartini (2018) | Reduce incidence of alcohol relapse in patients trying to achieve 6-month abstinence before liver transplantation listing  **Primary outcome:** Abstinence | **SC only - counselling (active comparator):** psychological addiction counselling within the transplantation centre/external treatment; sessions, format and content of sessions tailored to each patient; 8-week period; individual; licensed behavioural health clinicians (licensed psychiatrists or psychologists); concurrent pharmacotherapy not reported  **SMS-based RP plus SC:** SC plus RP (based on Marlatt & Gordon RP model: craving identification, mood, high-risk situation identification, coping strategies, trigger identification); 8 weeks: first four weeks 3 TM/day, last four weeks 3 TM/week; individual; SMS sent by research assistant; concurrent pharmacotherapy not reported | Not reported | **Biologically confirmed alcohol use rates - EtG/EtS positive (>500μg/l):** baseline SC 3/7 RP 2/8, week 8 SC 2/6 RP 0/8  **TLFB previous 30-day alcohol use:** baseline SC 1/6 RP 1/8, week 8 SC 0/6 RP 2/8 (p = 0.19)  **Alcohol craving:** not significant differences between conditions (p = 0.33) |
| Dieperink (2014) | Reduce alcohol use in patients with HCV and AUD  **Primary outcome:** Reduction | **EDU (active comparator):** health education sessions on general health (sleep hygiene, nutrition and diet, relaxation training and exercise); 4 sessions of 30-45 mins; 3 months; individual; 1 physician and 5 psychologists; one participant with two 30-day prescription of naltrexone.  **MET:** liver function and HCV-specific feedback, discussion of alcohol effects on HCV, liver fibrosis and antiviral therapy; 4 sessions of 30-45 mins; 3 months; individual; delivered by same staff; concurrent pharmacotherapy not reported. | **Drinks/week (SD):** baseline EDU 38.86 (46.19) MET 35.45 (25.42); 3 months EDU 19.94 (25.50) MET 13.35 (18.86); 6 months EDU 21.41 (29.39) MET 14.88 (27.94), mean difference between groups -6.52 (95% CI -16.9, 3.85), p = 0.57  **Heavy drinking days (≥4 drinks) (SD):** baseline EDU 15.04 (9.53) MET 14.03 (8.74); 3 months EDU 8.27 (8.98) MET 6.24 (9.31); 6 months EDU 7.38 (9.53) MET 5.10 (8.10), mean difference between groups -2.28 (95% CI -5.48, 0.93); p = 0.16  **% CDT > 2.1 (n):** baseline EDU 38.8 (19/49) MET 37.7 (20/53); 3 months EDU 36.6 (15/41) MET 30.6 (11/36); 6 months EDU 26.1 (12/46), MET 26.5 (9/34), mean difference between groups 0.4 % (95% CI -19.7, 20.4%); p = 0.97  **% EtG/EtS positive (n):** baseline EDU 79 (49/62) MET 73 (46/63); 3 months EDU 70.8 (34/48) MET 53.2 (25/47); 6 months EDU 58.9 (33/56) 55 MET (22/40), mean difference between groups 3.9% (95% CI -24.5, 16.6%); p = 0.70 | **% days abstinent (SD):** baseline EDU 34.6 (27.05) MET 34.98 (26.7); 3 months EDU 58.23 (34.05) MET 69.91 (31.9); 6 months EDU 59.49 (35.30) MET 73.15 (32.18), mean difference between groups 13.66 (95% CI 1.4,25.9); p < 0.024  **30-day abstinence, % (n):** 3 months EDU 13.3 (8/60) MET 24.1 (14/58); 6 months EDU 19.7(12/61) MET 25.4 (15/59), mean difference between groups 5.7% (95% CI -9.5, 21.0%); p = 0.45 |
| Kuchipudi (1990) | Attain sobriety  **Primary outcome:** Abstinence | **SC (active comparator):** assignment to ward team of attending physician, resident responsible for standard medical care during stay at acute medical unit  **MI:** interviews emphasizing need and benefits of alcoholism therapy and group discussions; 5 sessions; completed in 120 minutes; individual and group-based; medicine resident, principal nurse, gastroenterology fellow, social worker trained in alcoholism, psychiatric nurse therapy  Participants prescribed disulfiram (n = 1), antidepressants (n = 1) and benzodiazepines (n = 2) during outpatient evaluation | Not reported | **Sobriety, % (n):** SC 37.8 (19/51) MI 37.5 (21/56), no significant difference (p > 0.05) |
| Proeschold-Bell (2020) | Abstinence and relapse prevention  **Primary outcome:** Abstinence | **SBIRT (BI with MI) only:** AUDIT screening, 5-10min BI and MI intervention, referral to external alcohol treatment; delivered at every visit; individual; medical providers trained in SBIRT**;** concurrent pharmacotherapy not reported  **SBIRT (BI with MI) plus integrated alcohol treatment:** SBIRT and integrated HCV-alcohol treatment; up to 36 sessions; 6 months; individual and group therapy sessions (CBT, MET and substance abuse treatment), with telephone therapy available; addiction therapist, medical provider and psychiatrist; medication prescribed as appropriate | Not reported | **TLFB past 30-day full abstinence, % (n)**: 3 months SBIRT 19.0 (15/79) SBIRT + Alcohol Treatment 21.2 (18/85); 6 months SBIRT 20.5 (16/78) SBIRT + Alcohol Treatment 23.3 (20/86); 12 months SBIRT 26.4 (19/72) SBIRT + Alcohol Treatment 25.6 (21/82); 6-month intervention effect coefficient: 0.65 (95%CI -0.65,1.96), p = 0.33  **TLFB heavy drinking days per month (among patients who fully abstained for 30 days), mean (SD):** 7-12 months SBIRT 1.3 (1.8) SBIRT + Alcohol Treatment 1.1 (1.2); intervention effect coefficient: 1.1 (95%CI - 4.0,6.1), p = 0.68  **TLFB alcohol relapse measured by any heavy drinking days following 6-month abstinence, % (n):** 7-12 months SBIRT 26.7% (4/15) SBIRT + Alcohol Treatment 42.1% (8/19); intervention effect coefficient: 0.47 (95%CI -0.48,1.43), p = 0.33  **TLFB grams consumed per week (among patients who fully abstained for 30 days, mean, in g/week (SD): baseline SBIRT 186 (165) SBIRT + Alcohol treatment 206 (243)** 6 months SBIRT 94 (123) SBIRT + Alcohol Treatment 134 (246); 6 months intervention effect coefficient: 22.1g/week (95%CI -32.7, 76.9, p = 0.43), 12 months intervention effect coefficient 27.7g/week (95%CI -25.7, 81.1, p = 0.31) |
| Reid (2019) | Reduce alcohol consumption levels  **Primary outcome:** Reduction | **SC (active comparator):** review of overall health and liver disease, no formalised assessment or intervention for drinking reduction; 30min session; individual; nurse practitioner; concurrent pharmacotherapy not reported  **Combined BI with MI plus SC:** BI with MI and development of plan for drinking reduction; 10min of 30min session; individual; nurse practitioner; concurrent pharmacotherapy not reported | **TLFB 50% alcohol intake reduction, % (n)**: 4 weeks SC 40.9 (13) BI/MI 43.5 (10), p = 0.295; 8 weeks SC 42.9 (9) BI/MI 52.6 (10), p = 0.536  **AUD measured by AUDIT-C, mean (95% CI):** baseline SC 6.67 (5.66, 7.67) BI/MI 7.04 (6.08, 8.01); 4 weeks SC 6.11 (4.73, 7.49) BI/MI 5.24 (3.87, 6.61); 8 weeks SC 5.85 (4.58, 7.12) BI/MI 4.93 (3.67, 6.19); no significant differences between groups (p = 0.546)  **TLFB survey alcohol, mean (95% CI):** baseline SC 50.08 (33.34, 74.99) BI/MI 51.62 (34.86, 76.20); 4 weeks SC 33.74 (16.84, 66.29) BI/MI 12.35 (6.09, 24.12); 8 weeks SC 24.45 (10.37, 55.98) BI/MI 7.84 (2.98, 18.64), p = 0.069 | **Days where no alcohol was consumed, median:** baseline SC 19 (23.0) BI/MI 20 (17.0); 4 weeks SC 17.0 (15.5) BI/MI 24.0 (13.8); 8 weeks SC 16.0 (23.5) BI/MI 23.0 (17.5); no significant difference |
| Shen (2017) | Self-management of drinking behavior  **Primary outcome:** Abstinence | **SC (active comparator):** conventional treatment and health education; 10-15min; 12 weeks; individual; deputy chief nurse, psychological counsellor; concurrent pharmacotherapy not reported  **AA (peer support) plus SC:** conventional treatment and AA; weekly sessions; 10-15min; 12 weeks; individual and group based; deputy chief nurse, psychological counsellor; concurrent pharmacotherapy not reported | Not reported | **Relapse rate, % (n):** 3 months SC 36.84 (14) AA 16.22 (6), p < 0.05  **Alcohol dependence measured by MAST score (SD):** baseline SC 18.23 (1.86) AA 16.44 (1.47); 3 months SC 14.12 (1.50) AA 7.28 (0.96), p < 0.05  **Alcohol craving measured by PACS score (SD):** baseline SC 80.43 (2.33) AA 78.45 (6.56); 3 months SC 62.16 (3.24) AA 49.14 (8.29), p < 0.05 |
| Stein (2020) | Reduce drinking  **Primary outcome:** Reduction | **BA:** script reading on alcohol and HIV/HCV co-infection; 2 minutes; delivered at baseline study visit and during six phone sessions; 18 months; individual; licensed clinical psychologist; concurrent pharmacotherapy not reported  **Combined MI with MET:** MI and MET-based sessions; relapse prevention strategies worksheets made available by the interventionist and referral to medical care as needed; 20-30 minutes; six sessions; 18 months; individual; licensed clinical psychologist; concurrent pharmacotherapy not reported | **TLFB IRR alcohol use days (p):** 3 months BA 0.78 (0.005) MI/MET 0.63 (0.045); 6 months BA 0.72 (< 0.001) MI/MET 0.66 (0.049); 9 months BA 0.65 (< 0.001) MI/MET 0.48 (< 0.001); 12 months BA 0.65 (0.001) MI/MET 0.48 (< 0.001); 15 months BA 0.61 (< 0.001) MI/MET 0.48 (< 0.001); 18 months BA 0.68 (0.001) MI/MET 0.50 (< 0.001); 24 months BA 0.61 (< 0.001) MI/MET 0.47 (< 0.001); between group differences not statistically significant.  **TLFB IRR mean drinks/day (p):** 3 months BA -1.44 (0.147) MI/MET -2.20 (0.001); 6 months BA -2.37 (0.002) MI/MET -1.76 (< 0.001); 9 months BA -0.64 (0.698) MI/MET -3.21 (< 0.001); 12 months BA -2.94 (0.003) MI/MET -3.32 (< 0.001); 15 months BA -3.39 (< 0.001) MI/MET -2.57 (< 0.001); 18 months BA -3.77 (< 0.001) MI/MET -1.81 (0.043); 24 months BA -3.66 (< 0.001) MI/MET -2.90 (< 0.001); between group differences not statistically significant. | Not reported |
| Weinrieb (2011) | Reduce risk of post transplantation drinking by addressing AUD before transplantation  **Primary outcome:** Reduction | **SC (active comparator):** referral to outpatient therapy and community AA/NA meetings; 6 months; individual and group-based; concurrent pharmacotherapy not reported  **MET:** MET and encouragement to attend AA meetings (50min) and referral to case management; 4-7 sessions; 6 months; individual and group-based; addiction therapist; concurrent pharmacotherapy not reported | **TLFB total number of drinks, median (IQR) at longest FU:** SC 59 (8.0,136.0) MET 7.0 (2,8); beta = -2.51 (95%C -4.14, -0.87, p = 0.003)  **TLFB total number of drinking days, median (IQR):** SC 7.0 (2.0-11.0) MET 2.0 (1.0-2.0), beta = -2.62 (95% -4 .40, - 0.83, p = 0.004)  **TLFB drinks per drinking day, median (IQR):** MET 3.5 (2.0-5.0) SC 4.3 (3.4-8.0); beta = 0.70 (95%C -1.36, - 0.04, p = 0.036) | Not reported |
| Willenbring & Olson (1999) | Induce remission of drinking, frequency and severity of relapse and improve related health conditions  **Primary outcome:** Abstinence | **SC (active comparator):** outpatient and inpatient consultation, treatment, and referral to general and specialty medical care clinics; medical staff; concurrent pharmacotherapy not reported  **IOT (combined CBT with MET):** comprehensive medical care and development of treatment plan and motivation to change discussions, with mental health, social services and intensive alcohol treatment provided as needed; visit frequency subject to medical management; individual and possible involvement of family members; physicians and nurse practitioners; medication prescribed as appropriate, but none reported | Not reported | **TLFB days since last drink, mean:** baseline SC 2.5 IOT 2.0; 24 months SC 16.5 IOT 139 Control 16.5, p = 0.08  **TLFB abstinent patients, % (n):** IOT 74% (28) Control 48% (18), p = 0.02  **TLFB past 30-day drinking days, mean (SD):** baseline SC 14.4 (12.3) IOT 15.8 (12.3); 24 months SC 7.0 (10.0) IOT 3.7 (7.9), p = 0.03  **TLFB drinks per drinking day, mean (SD):** baseline SC 8.9 (7.9) IOT 11.1 (10.3); 24 months SC 3.0 (4.5) IOT 1.8 (3.7), p = 0.05  **ASI alcohol rating (SD):** baseline SC 0.33 (0.26) IOT 0.39 (0.22); 24 months SC 0.19 (0.19) IOT 0.14 (0.19) control p = 0.27 |
| Zule (2009) | Eliminate alcohol use  **Primary outcome:** Abstinence | **EDU (active comparator):** two cue-card and four video-based sessions; participants rewarded $10 at last 3 sessions; duration of sessions ranging from 20 to 78 minutes; individual; concurrent pharmacotherapy not reported  **MI:** two cue-card and four motivational sessions; participants rewarded $10 each session; duration ranging from 19 to 41 minutes; individual; conducted by lay person from community trained in motivational interviewing; concurrent pharmacotherapy not reported  Both groups offered testing for HCV, HIV and hepatitis B | Not reported | **Past 30-day alcohol use, %:** participants in MI group less likely to be drinking at 6 months (OR 0.67, 95% CI 0.46, 0.97, p = 0.035); no significant difference at 12-month follow-up |

* As described by study authors

% CDT: percentage of carbohydrate-deficient transferrin; %CDT: percentage carbohydrate-deficient transferrin; AA: Alcoholics Anonymous; AUD: alcohol use disorder; AUDIT- C: alcohol use disorders identification test – consumption; AUDIT: alcohol use disorders identification test; BA: brief advice; BI: brief intervention; CBT: cognitive behavioural therapy; CI: confidence interval; EDU: control health education; EtG: ethyl glucuronide; EtS: ethyl sulfate; HCV- PERF: hepatitis C psychoeducation responsive to families; HCV- SMP: hepatitis C self-management program; HCV: hepatitis C virus; HIV: human immunodeficiency virus; IOT: integrated outpatient treatment; IQR: interquartile range; IRR: incidence rate ratio; MAST: Michigan alcohol screening test; MET: motivational enhancement therapy; MI: motivational interviewing; NA: Narcotics Anonymous; PACS: Parnia Alcohol Craving Scale; p: p-value; PEth = phosphatidylethanol; SBIRT: screening, brief Intervention, and referral to treatment; SD: standard deviation; TLFB: timeline followback; TM: text message

***Legend: summary of intervention goal, primary outcome, study conditions, reduction, and abstinence outcomes across the studies.***

**Supplementary Table 4: Summary of evidence on reduction outcomes**

| **Study** | **Intervention vs Control** | **Condition effect** | | **Overall effect of intervention on reduction outcomes at longest FU** |
| --- | --- | --- | --- | --- |
| Dieperink *et al.* (2014) | MET vs EDU | No statistically significant difference between groups in drinks/week (p = 0.57), heavy drinking days (p = 0.16), %CDT (p = 0.97) and % EtG/EtS positive (p = 0.70) | Not significant | |
| Proeschold-Bell  *et al.* (2020) | SBIRT (BI with MI) only vs SBIRT (BI with MI) plus integrated alcohol treatment | No statistically significant difference between groups at 6 (p = 0.43) and 12 months (p = 0.31) | Not significant | |
| Reid  *et al.* (2019) | Combined BI with MI plus SC vs SC only | 50% reduction in alcohol consumption greater with intervention (53%) compared to control (43%) but not significant (p = 0.536) | Not significant | |
| Stein  *et al.* (2020) | Combined MI with MET vs BA | No statistically significant difference between groups | Not significant | |
| Weinrieb  *et al.* (2011) | MET vs SC | Reduction greater in intervention group in total number of drinks (beta = - 2.51, 95% CI -4.14, 0.087, p = 0.003), drinking days (beta = -2.62, 95% CI -4.40, -0.83 p = 0.004) and drinks per drinking day (beta = -0.70, 95% CI -1.36, - 0.04, p = 0.035) | Significant | |

***Legend: summary of results for the efficacy of psychosocial interventions in reduction outcomes.***

**Supplementary Table 5: Summary of evidence on abstinence outcomes**

| **Study** | **Intervention vs Control** | **Condition effect** | **Overall effect of intervention on abstinence outcomes at longest FU** |
| --- | --- | --- | --- |
| DeMartini *et al.* (2018) | SMS-based RP vs SC (counselling) | No statistically significant difference between groups (p = 0.19) | Not significant |
| Dieperink et al. (2014) | MET vs EDU | Significant increase in % of days abstinent in intervention group (p = 0.024) | Significant |
| Kuchipudi *et al.* (1990) | MI vs SC | No statistically significant difference between groups (p > 0.05) | Not significant |
| Proeschold-Bell *et al.* (2020) | SBIRT (BI with MI) plus integrated alcohol treatment vs SBIRT only | No statistically significant difference between groups (intervention effect 0.65, 95% CI -0.65, 1.96, p = 0.33) | Not significant |
| Reid *et al.* (2019) | Combined BI with MI plus SC vs SC only | No statistically significant difference between groups | Not significant |
| Shen *et al.* (2017) | Peer support (AA) vs SC | Significant reduction in relapse rate in intervention group (p < 0.05) | Significant |
| Willenbring & Olson *et al.* (1999) | IOT (combined CBT with MET) vs SC | Significant increase in abstinence in intervention group (p = 0.02) | Significant |
| Zule, *et al.* 2009 | MI vs EDU | Significant difference between groups (OR = 0.67, 95% CI 0-46, 0.97, p = 0.035) at 6 months, but not sustained at 12 months | Not significant |

***Legend: summary of results for the efficacy of psychosocial interventions in abstinence outcomes.***

**
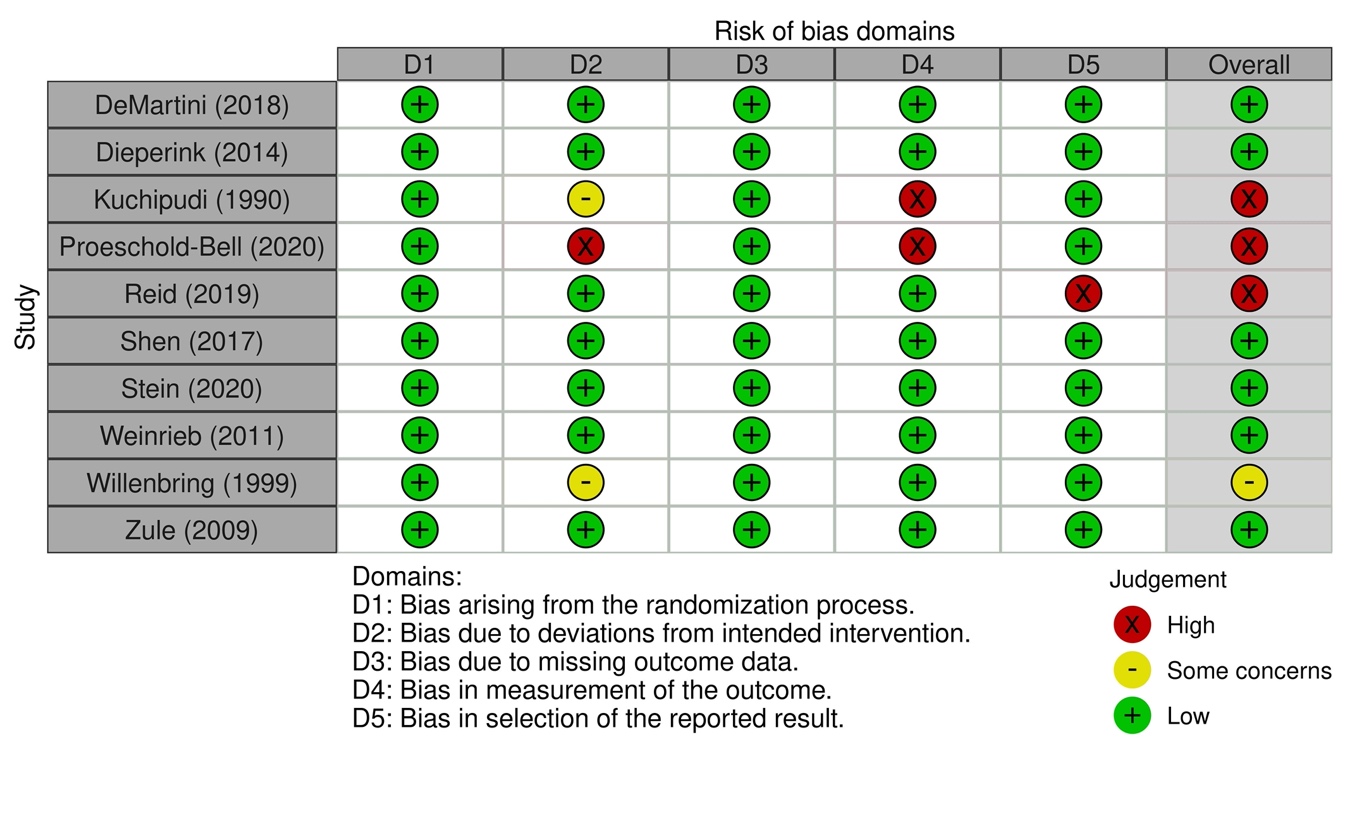
Supplementary Figure 1: RoB2 assessment by domain.**

***Legend:***  ***Traffic light plot with domain-level and overall risk of bias judgements.***

**Supplementary Figure 2: Rob2 assessment with domain distribution.**

**
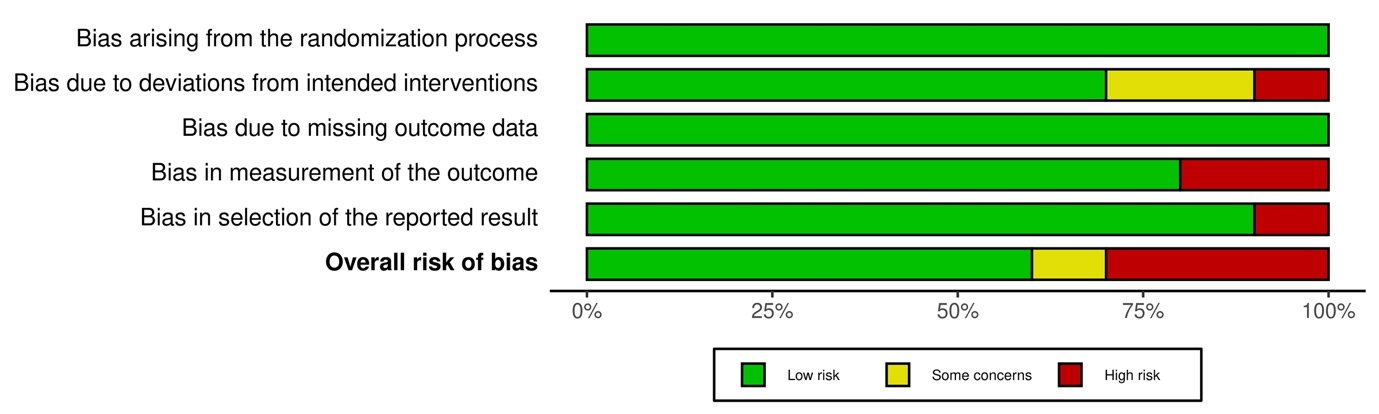
**

***Legend: Weighted bar plot with bias distribution within each domain.***

**Supplementary Table 6: GRADE certainty of evidence assessment of reduction outcomes**

| **Outcome and participants** | **Methodological limitations** | **Indirectness** | **Imprecision** | **Inconsistency** | **Publication bias** | **Overall certainty** |
| --- | --- | --- | --- | --- | --- | --- |
| c | Two studies (Reid *et al.*, 2019; Proeschold-Bell *et al.*, 2020) were classified as high risk of bias, the remaining three studies (Weinrieb *et al.*, 2011; Dieperink *et al.*, 2014; Stein *et al.*, 2020) were of low risk of bias.  Serious | Participants, interventions, comparators and outcome measures across studies directly informed the aims of the review; interventions were compared indirectly downgrading the rating of this domain.  Not serious, borderline | Four of the studies (Dieperink *et al.*, 2014; Reid *et al.*, 2019; Proeschold-Bell *et al.*, 2020; Stein *et al.*, 2020) did not detect a statistically significant difference between groups and presented wide confidence intervals, possibly linked to the small number of participants enrolled.  Serious | The included studies had substantial clinical (participants, interventions, and outcomes) and methodological (study design) heterogeneity, leading to a moderate inconsistency across the overall effect of the interventions across reduction outcomes.  Not serious, borderline | No suspicions of publication bias as search strategy was comprehensive and detected both negative and positive, small and large-scale trials, and these have been included in the review.  Not suspected | ⨁⨁⨁◯ Moderate certainty |

***Legend: Grade certainty of evidence assessment for reduction outcomes. Overall certainty - moderate: the authors believe that the true effect is probably close to the estimated effect.***

**Supplementary Table 7: GRADE certainty of evidence assessment of abstinence outcomes**

| **Outcome and participants** | **Methodological limitations** | **Indirectness** | **Imprecision** | **Inconsistency** | **Publication bias** | **Overall certainty** |
| --- | --- | --- | --- | --- | --- | --- |
| Abstinence  n = 1317 (8 RCTs) | Three of studies were of high risk of bias (Kuchipudi *et al.*, 1990; Reid *et al.*, 2019; Proeschold-Bell *et al.*, 2020), one presented some concerns (Willenbring and Olson, 1999) and the remaining four presented a low risk of bias (Zule *et al.*, 2009; Dieperink *et al.*, 2014; Shen *et al.*, 2017; DeMartini *et al.*, 2018)  Serious | Participants, interventions, comparators and outcome measures across studies directly informed the aims of the review; interventions were compared indirectly downgrading the rating of this domain.  Not serious, borderline | Five of the studies (Zule *et al.*, 2009; Dieperink *et al.*, 2014; Shen *et al.*, 2017; DeMartini *et al.*, 2018) did not detect significant differences between the intervention and control groups and also presented large confidence intervals.  Serious | The included studies had substantial clinical (participants, interventions, and outcomes) and methodological (study design) heterogeneity; nevertheless, four studies consistently favoured the effect of the intervention across abstinence outcomes (Willenbring and Olson, 1999; Dieperink *et al.*, 2012; Shen *et al.*, 2017; DeMartini *et al.*, 2018).  Not serious, borderline | No suspicions of publication bias as search strategy was comprehensive and detected both negative and positive, small and large-scale trials, and these have been included in the review.  Not suspected | ⨁⨁⨁◯ Moderate certainty |

***Legend: Grade certainty of evidence assessment for reduction outcomes. Overall certainty - moderate: the authors believe that the true effect is probably close to the estimated effect.***
